# Marine cyanobacterium *Spirulina maxima* as an alternate to the animal cell culture medium supplement

**DOI:** 10.1038/s41598-021-84558-2

**Published:** 2021-03-01

**Authors:** Younsik Jeong, Woon-Yong Choi, Areumi Park, Yeon-Ji Lee, Youngdeuk Lee, Gun-Hoo Park, Su-Jin Lee, Won-Kyu Lee, Yong-Kyun Ryu, Do-Hyung Kang

**Affiliations:** 1 Jeju Marine Research Center, Korea Institute of Ocean Science and Technology (KIOST), Jeju, Republic of Korea; 2 School of Pharmacy, Sungkyunkwan University, Seoul, Republic of Korea; 3 Department of Ocean Science, University of Science and Technology (UST), Jeju, Republic of Korea

**Keywords:** Biotechnology, Cell biology

## Abstract

Serum is a stable medium supplement for in vitro cell culture. Live cells are used in stem cell research, drug toxicity and safety testing, disease diagnosis and prevention, and development of antibiotics, drugs, and vaccines. However, use of serum in culture involves concerns such as an ethical debate regarding the collection process, lack of standardized ingredients, and high cost. Herein, therefore, we evaluated the possibility of using edible cyanobacterium (*Spirulina maxima*), which is a nutrient-rich, sustainable, and ethically acceptable source, as a novel substitute for fetal bovine serum (FBS). H460 cells were cultured to the 10th generation by adding a mixture of spirulina animal cell culture solution (SACCS) and FBS to the culture medium. Cell morphology and viability, cell cycle, apoptosis, proteomes, and transcriptomes were assessed. We observed that SACCS had better growth-promoting capabilities than FBS. Cell proliferation was promoted even when FBS was replaced by 50–70% SACCS; there was no significant difference in cell shape or viability. There were only slight differences in the cell cycle, apoptosis, proteomes, and transcriptomes of the cells grown in presence of SACCS. Therefore, SACCS has the potential to be an effective, low-cost, and eco-friendly alternative to FBS in in vitro culture.

## Introduction

In vitro cell culture is widely used in various research fields, especially in the pharmaceutical and biotechnological industries. Fetal bovine serum (FBS) is generally used as a cell culture supplement to optimize cell culture conditions. FBS is a complex composite of high- and low-molecular-weight biomolecules and mainly consists of vitamins, hormones, minerals, factors (for cell growth, attachment, and spreading), and transport proteins^1,2^. However, the use of FBS involves certain concerns. FBS is collected in aseptic conditions by separating the fetus from the slaughtered pregnant cow and stabbing large diameter needles into the heart of the unanesthetized fetus^3^. Therefore, there is a possibility of FBS contamination by viruses, mycoplasma, and prions and its use may involve both ethical issues with the collection process and scientific issues with variations in composition between batches^4–7^. Additionally, FBS prices have tripled over the past few years owing to increased demand^8^. For these reasons, a major focus of cell culture research has been to identify or develop FBS alternatives. Although there have been attempts to replace FBS with serum from other animals (goats, pigs, or horses), their application was limited, as they helped the growth of only some cell lines^9–12^.

*Spirulina* (*Arthrospira*) *maxima* is a helicoidal, unbranched, and filamentous cyanobacterium belonging to the *Oscillatoriaceae* family^13^. *S. maxima* is a rich source of proteins (~ 60–70% of its dry weight), phycobiliprotein, vitamins, essential fatty acids, carotenoids, and minerals^14,15^. It also contains antioxidants that can protect cells from oxidative damage. *S. maxima* is recognized as an unrestricted food by the Korea Food and Drug Administration and a Generally Recognized As Safe substance, indicating its non-toxic nature^4^. Several pharmaceutical companies produce *Spirulina* and its products, which are sold as food supplements in several health food stores worldwide. Recently, research on the therapeutic effects of *S. maxima* has attracted great attention. Several clinical studies have suggested various therapeutic effects ranging from radiation protection, increase in intestinal lactobacillus abundance, alleviation of cholesterol levels and cancer, strengthening of the immune system, and lowering of nephrotoxicity caused by heavy metals and drugs^16–18^. In addition, previous in vitro and in vivo studies have reported the anti-oxidant, anticancer, anti-hyperlipidemic, anti-neurotoxic, and anti-type 1 diabetic effects of *S. maxima*^19–23^. However, the complete nutritional, immunological, and physiological functions of *Spirulina* remain unknown owing to a lack of diverse and advanced approaches. Hence, there is a need to develop novel technologies to study the practical applications of marine *Spirulina*-derived medicines and biomaterials.

As *S. maxima* contains components such as proteins, minerals, trace elements, and lipids that assist in cell growth and proliferation, it has a higher protein content than commonly used *Spirulina platensis*^24^, it has the potential to replace FBS as a cell culture supplement. This strain, unlike land crops, contains many nutritional ingredients that can exert multiple effects on cell lines in a single extract. The current study aimed to investigate the extraction, physical properties, biocompatibility, and range of use of *S. maxima* extract for its application as an FBS alternative.

## Results

### Characterization and contamination detection of SACCS

**Figure 1:**
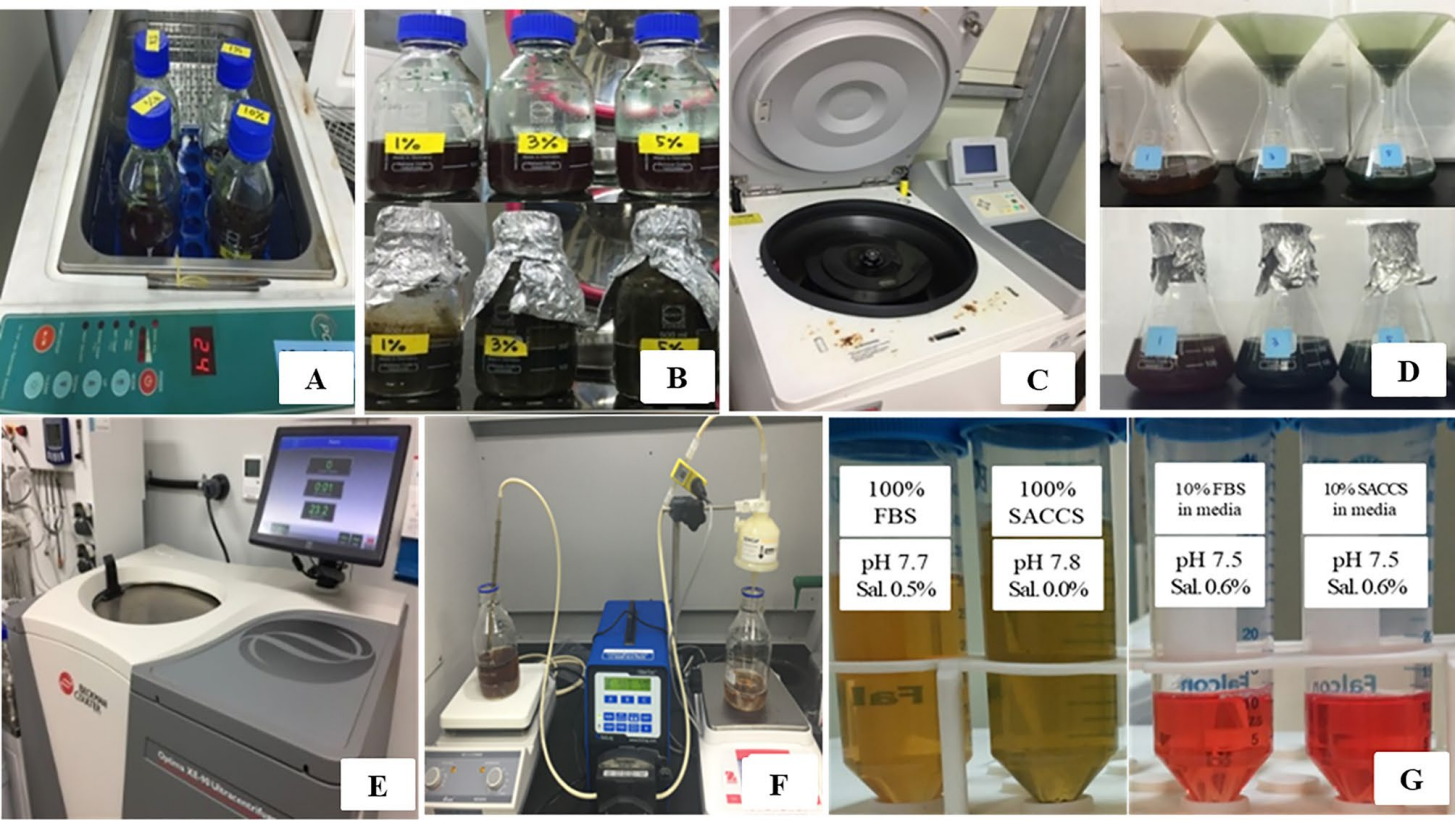
Schematic manufacturing process for spirulina animal cell culture solution (SACCS). (**A**) Cell disruption through ultrasonication (**B**) Extraction and sterilization through high temperature and high-pressure treatment. (**C**) Centrifugation. (**D**) Supernatant recovery using filter paper. (**E**) Ultracentrifugation. (**F**) 0.2-μm sterilization pump filter system. (**G**) Comparison of characteristics between fetal bovine serum (FBS) and SACCS [pH and salinity (Sal.)] in each cocktailed media.

**Figure 2:**
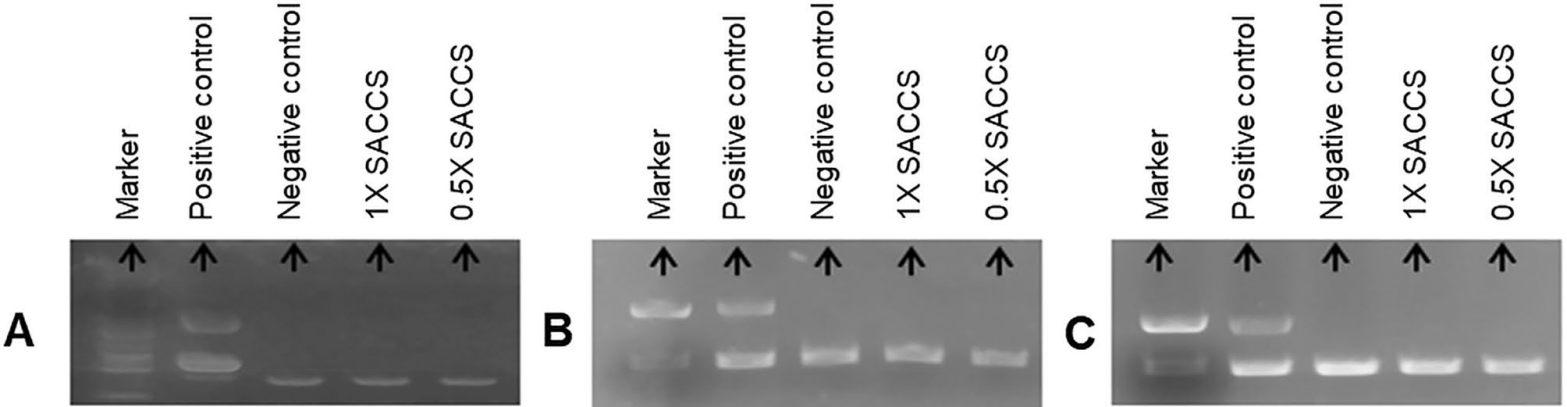
Microbial contamination test on SACCS. Polymerase chain reaction (PCR) experiments for the detection of (**A**) mycoplasma, (**B**) fungal, and (**C**) bacterial contamination in the SACCS samples.

Salinity is a key factor that regulates the growth of organisms and preserves their cellular structure. In addition, cellular growth also requires an optimal and stable pH. Hence, the salinity and pH of SACCS and FBS were measured and compared. The pH and salinity of FBS were 7.7 and 0.5%, respectively and those of SACCS were 7.8 and 0%, respectively (Fig. 1G). However, both pH and salinity were not different when FBS and SACCS were each mixed in MEM medium at a 10% ratio (Fig. 1G). Contamination with bacteria, fungi, endotoxins, and mycoplasma can have detrimental effects on the cell culture. To confirm the complete removal of contamination source(s), the contamination levels were assessed by PCR (Fig. 2), and the absence of fungi, bacteria, and mycoplasma was confirmed (Fig. S1).

Table 1 shows the general components, minerals, and heavy metal content of SACCS and FBS analyzed in this study. The SACCS contained 13.20% of carbohydrate, 79.20% protein, and 4.40% lipid, compared with FBS contained 8.08% of carbohydrate, 85.00% of protein, and 1.75% of lipid. Their main biochemical component was protein, and a similar ratio was has been confirmed. The mineral contents were determined to have a similar ratio to each other, and only a small amount of heavy metals were detected in the SACCS. It was found to have not completely similar, but the overall component contents ratio has similar properties.

**Table1:** Summary of biochemical components of SACCS and FBS. SACCS: 1 × SACCS in 99% distilled water.

Organic and inorganic indices	Components	SACCS		FBS	
		Concentration (mg/L)	Percentage (%)	Concentration (mg/L)	Percentage (%)
Biochemical components	Carbohydrate	900.00	13.20	3700.00	8.08
	Protein	5400.00	79.20	38900.00	85.00
	Lipid	300.00	4.40	800.00	1.75
Minerals	Calcium	17.00	0.25	136.60	0.30
	Sodium	85.00	1.25	2164.30	4.73
	Magnesium	6.40	0.09	39.10	0.09
	Potassium	10.00	0.15	6.10	0.01
	Manganese	0.10	0.00	0.00	0.00
	Iron	7.90	0.12	3.20	0.01
	Copper	1.30	0.02	0.40	0.00
	Boron	0.40	0.01	0.40	0.00
	Phosphorus	89.10	1.31	12.30	0.03
	Zinc	1.10	0.02	3.50	0.01
Heavy metals	Lead	0.02	0.00	0.00	0.00
	Cadmium	0.01	0.00	0.00	0.00
	Mercury	0.00	0.00	0.00	0.00

### Efficacy of SACCS as an alternative for FBS in cell culture

**Figure 3:**
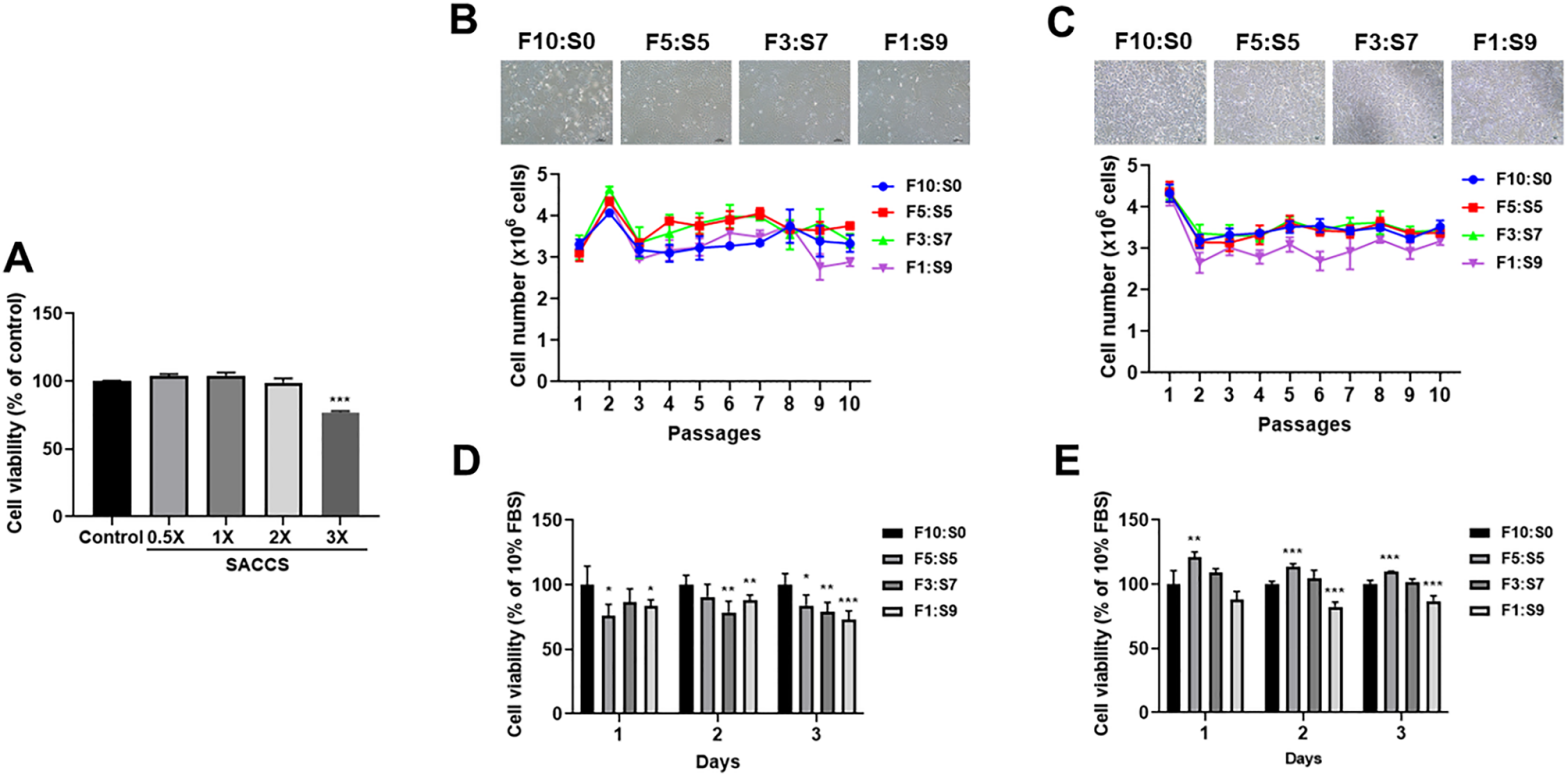
Evaluation of FBS substitution efficacy of SACCS. Cytotoxicity test of distilled water (DW), fetal bovine serum (FBS), 0.5× , 1× , 2 × , and 3 × spirulina animal cell culture solution (SACCS) on human lung cancer cell line H460 (A). Comparison of the cell morphology, metabolic proliferation activities, and cell viabilities of the cells in the control medium (FBS) and SACCS-substituted medium [1× SACCS (B, D) and 0.5× SACCS (C, E)]. Data are expressed as mean ± SD of three independent experiments. *p < 0.05; **p < 0.01; ***p < 0.001 compared with the cells cultured in F10:S0. Bar = 100 μm. Figures were made with GraphPad Prism (ver. 8.1.1, San Diego, USA), https://www.graphpad.com/scientific-software/prism/.

Cytotoxicity of SACCS was determined by testing viability of H460 cells treated with 0.5×, 1×, 2×, and 3× SACCS using a WST assay kit. The viability of cells grown in medium containing up to 2× SACCS was found to be 100% or more; cytotoxicity was not observed (Fig. 3A). Two SACCS concentrations (0.5× and 1×) were combined with FBS in various ratios (F5:S5, F3:S7, and F1:S9) and added as an FBS alternative in the culture medium. The number of cells was counted and photographed with a microscope at each passage. The results for 10 passages with H460 cells are depicted in Fig. 3. When 1× SACCS was used, the cell growth rate was confirmed to be 112% in F5:S5, 102% in F3:S7, and 87% in F1:S9 compared with that in F10:S0 (Fig. 3B). There was no significant change in the shape of the cells. In case of 0.5× SACCS, although fluctuations were noted, the cells in F5:S5-containing medium showed growth rates similar to those in F10:S0-containing medium during the 10 passages. In contrast, cells in F1:S9 exhibited constant lower cell numbers compared with the control (*p* < 0.001). F3:S7 showed approximately 97% efficacy (Fig. 3C). FBS and SACCS mixture supported cell growth throughout the 10 passages of subculture at all the mixing ratios. In terms of cell morphology, the cells cultured in F5:S5 were similar to those in F10:S0, but cells in F1:S9 revealed cytoplasmic vacuolization or blurring of intercellular boundaries.

To detect the biological effects of the supplement on cell proliferation, cells subcultured for 10 generations were transferred to a 96-well plate and the cell viability was detected for 3 days using WST assay. When 1× SACCS was used, the cell survival rate was not more than 100% in all the mixing ratios (Fig. 3D). However, when 0.5× SACCS was used, cell viability was confirmed to be 100% or more till 70% SACCS replacement (Fig. 3E).

**Figure 4:**
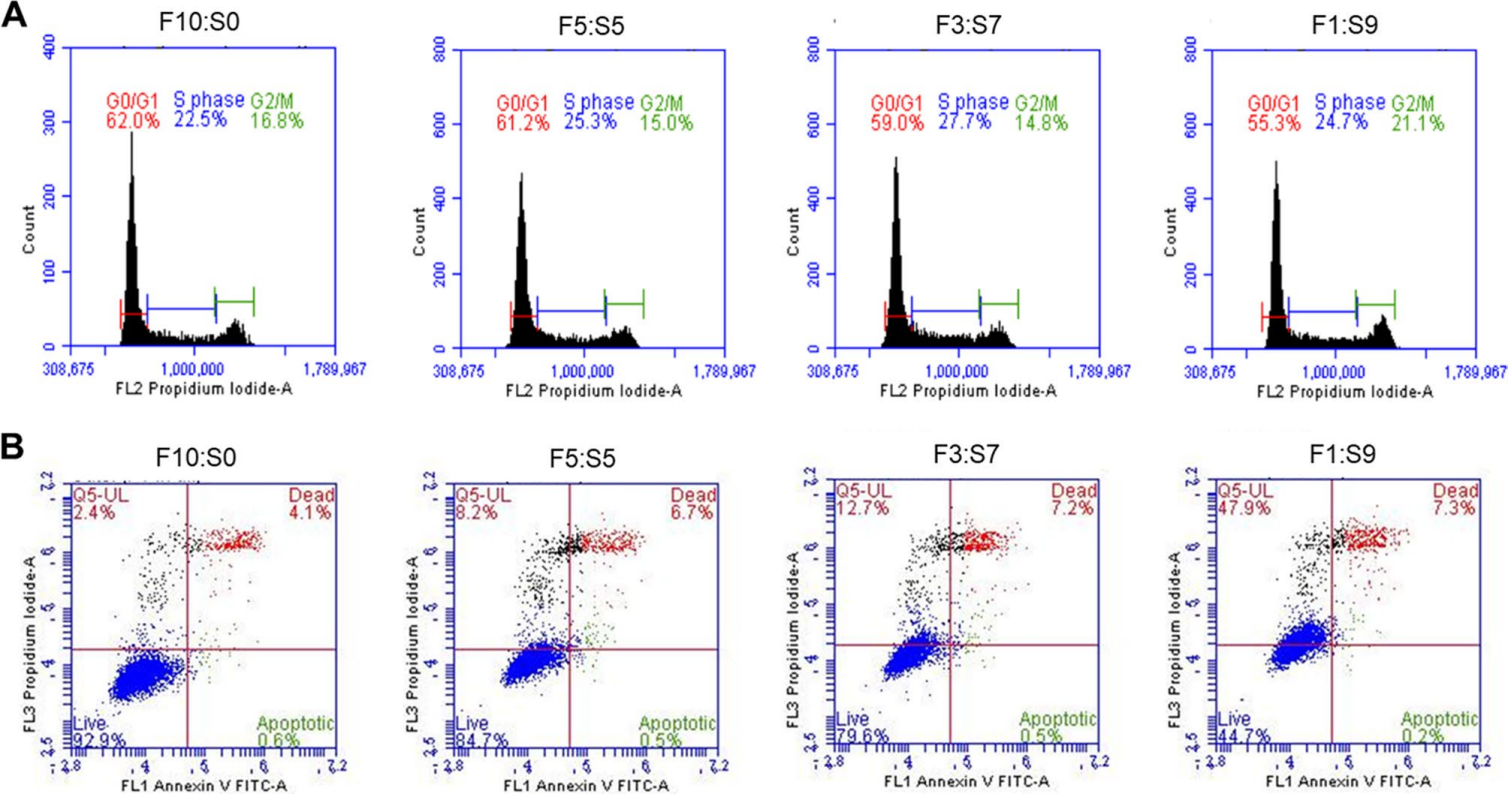
Cell cycle and apoptotic cell death type distribution of the 10-passage adapted H460 cell line in control medium (FBS) and 0.5× SACCS-substituted medium. Cells were stained with Annexin V-FITC and PI and analyzed on a BD FACS Accuri C6 flow cytometer. Distribution of (**A**) percentage of cell cycle phases and (**B**) apoptosis in media with different FBS:SACCS ratios.

The normal cell cycle is classified into G_1_, S, G_2_, and M phases, where cell growth occurs in the G_1_ phase, DNA replication in the S phase, growth and preparation for the cell division in the G_2_ phase, and cell division in the M phase^25^. We compared and analyzed the cell cycle phases of the cells cultured in SACCS-supplemented media with that of the control. The cell cycles of H460 cultured for a long time in FBS control and F5:S5 and F3:S7 media were almost similar. However, the ratio of G_0_ and G_1_ phase was decreased while the ratio of G_2_ and M phase was increased in the cells cultured in F1:S9 (Fig. 4A).

To further determine whether an increase in SACCS ratio in the medium induced cell growth inhibition and apoptosis in H460 cells, cell death was analyzed by staining with annexin V-FITC and PI, followed by flow cytometer. The results showed 4.7% apoptosis induced in the FBS control, but 7.2%, 7.7%, and 7.5% apoptosis induced in the F5:S5, F3:S7, and F1:S9 groups, respectively. In case of the cells cultured in SACCS culture medium, the apoptosis rates did not change significantly (Fig. 4B).

### Transcriptomic and proteomic analyses

**Figure 5:**
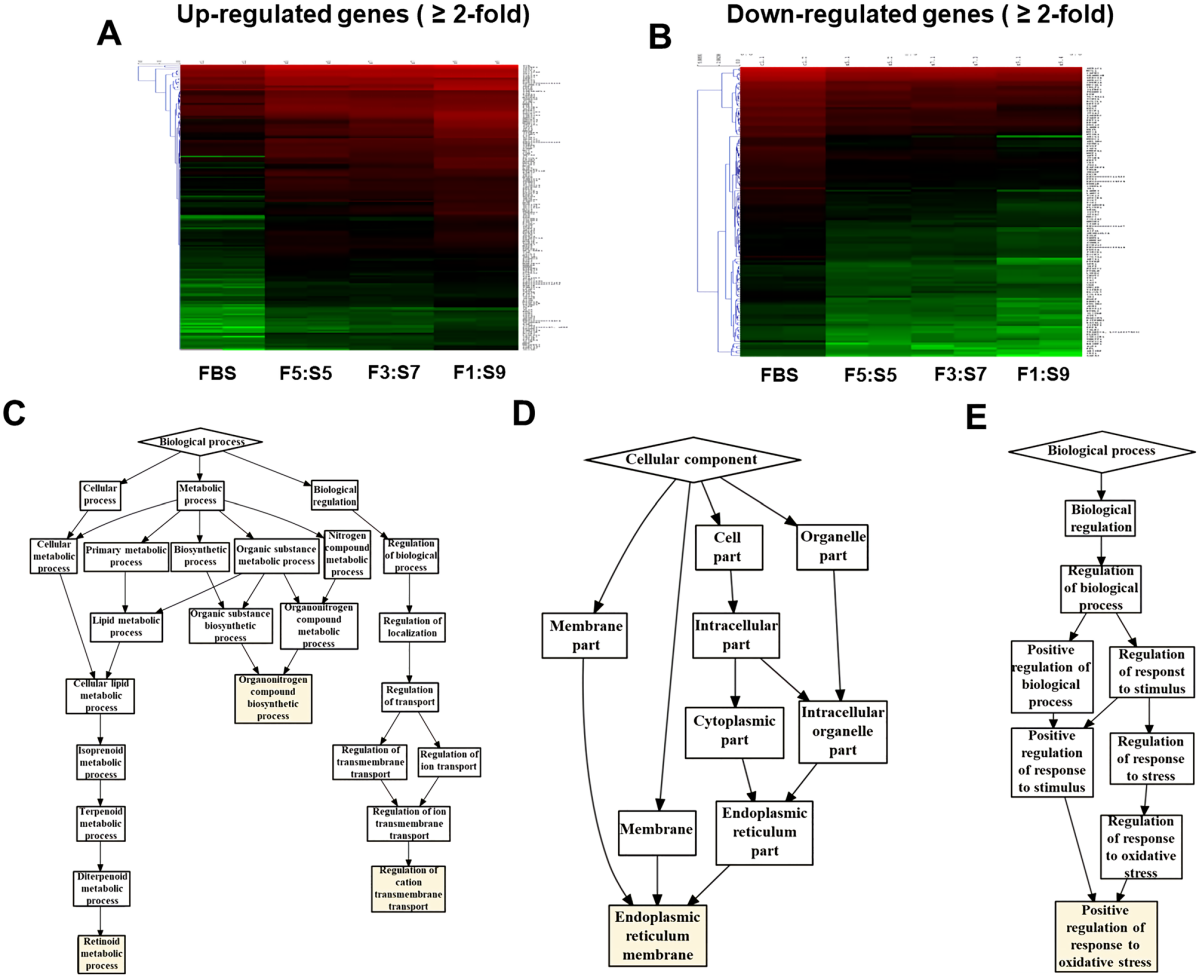
Transcript analysis of SACCS-adapted H460 cell line. Heat map of differentially expressed genes with more than twofold expression—(**A**) up-regulated genes and (**B**) down-regulated genes. Statistically significant (*p* ≤ 0.001) differentially expressed gene ontology enrichment analysis of cells cultured in (**C**) F5:S5, (**D**) F3:S7, and (**E**) F1:S9 media. Sample libraries were filtered onto the assembled transcriptome using Bowtie2 (ver. 2.4.2), http://bowtie-bio.sourceforge.net/bowtie2/index.shtml. Differentially expressed genes (DEGs) with a fold change of 2 or more (*p* value < 0.05) were mapped and visualized (**A** and **B**) by the CLRNAseq program (ver. 1.00.06, Chunlab, Seoul, Korea), https://www.chunlab.com/ngs/eng/service/rna. Gene ontology was analyzed using GOrilla for the associated genes (**C**, **D** and **E**), http://cbl-gorilla.cs.technion.ac.il.

**Figure 6:**
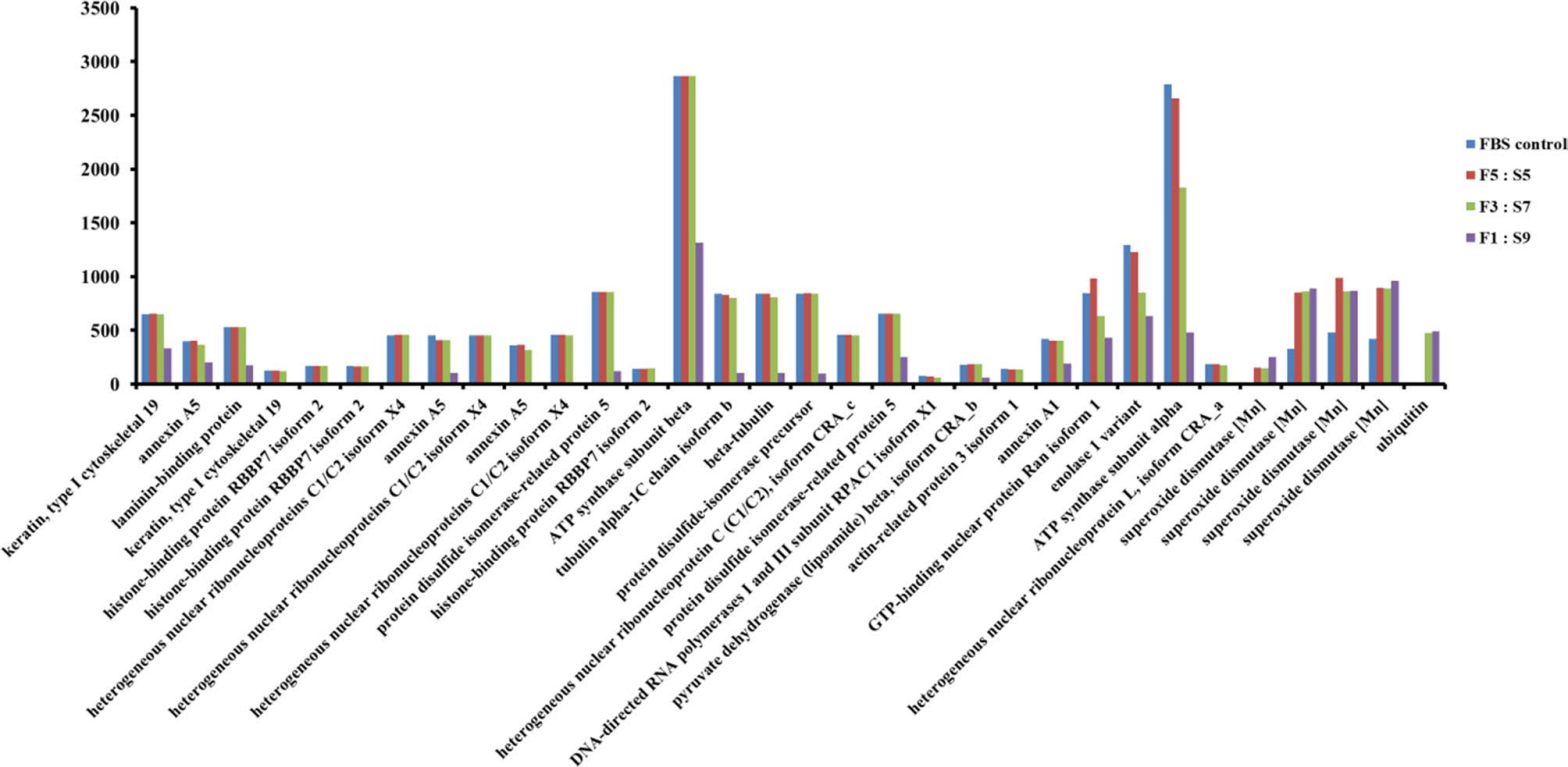
Proteomic analysis of SACCS-adapted H460 cell line. Bar diagrammatic representations of the differentially expressed proteins (*p* ≤ 0.05) identified in 2-DE are displayed. Spots were detected and quantified using PDQuest software (ver. 7.0, Bio-Rad, Hercules, USA), https://www.bio-rad.com/ko-kr/product/pdquest-2-d-analysis-software?ID=966deb78-2656-437f-b7a4-ab0a9bd45c8d.

For gene expression profile analysis, we generated more than 45 million mRNA reads per sample. A total of 48.4 (control), 48.1 (F5:S5), 46.8 (F3:S7), and 46.2 (F1:S9) million reads were obtained for H460 cells and could be mapped uniquely to the respective reference genomes (Fig. 5A,B). Compared with the control group, we identified 182, 152, and 272 DEGs in the F5:S5, F3:S7, and F1:S9 groups, respectively. Furthermore, 100 up-regulated and 82 down-regulated genes were observed in F5:S5, 73 up-regulated and 79 down-regulated genes in F3:S7, and 142 up-regulated and 130 down-regulated genes in F1:S9. Forty-nine DEGs were identified in all the three experimental groups. In addition, GO enrichment analysis for each sample in the biological process category revealed that F5:S5 included organonitrogen compound biosynthetic process (GO: 1901566), retinoid metabolic process (GO: 0001523), and regulation of cation transmembrane transport (GO: 1904062). Among them, most DEGs were those involved in retinoid metabolic process (Fig. 5C). In the F3:S7 and F1:S9 groups, the endoplasmic reticulum membrane (GO: 0005789) in the cellular component category (Fig. 5D) and the positive regulation of response to oxidative stress (GO: 1902884) in the biological process category (Fig. 5E) were found to be significantly enriched GO terms, respectively. Proteomic analysis of SACCS-adapted H460 cell line by 2D-PAGE revealed 76 protein spots showing differential expression. In the F5:S5 group, eight protein spots showed > twofold increase in expression, whereas in the F3:S7 group, the expression of nine protein spots was increased and that of eight spots was decreased. Finally, in the F1:S9 group, expression of five proteins was increased and expression of 48 proteins was decreased. We identified the amino acid sequences of the five increased spots and the 30 decreased protein spots showing a change in expression over two-fold compared with that in the control. The relative expression changes of 30 proteins with more than two-fold decrease in expression were not significantly different in all proteins except for five proteins in the F5:S5 and F3:S7 groups, and proteins were reduced or not expressed in only F1:S9 group. Of the five proteins whose expression was more than twice the expression of that in F10:S0, four protein spots were identified as superoxide dismutase protein and the remaining one was identified as ubiquitin. Hence, as the ratio of SACCS increased, the expression of antioxidant enzyme superoxide dismutase tended to increase (Fig. 6).

## Discussion

In this study, the effects of SACCS as a serum substitute on cell culture were systematically evaluated by studying cell proliferation, morphology, overall survival rate, cell cycle, apoptosis, and carrying out proteomic and transcriptomic analysis. First, a contamination test was performed to verify the physicochemical properties of SACCS and the reliability of the cell culture test. Contamination by bacteria, fungi, and mycoplasma adversely affects cell line growth, cell properties, and cell functions, and may cause changes in the experimental results^26,27^. Through the contamination test of SACCS, factors that could affect the cell culture test results were minimized. In addition, components and physicochemical factors of SACCS were analyzed to compare similarities with FBS. Osmotic and pH imbalances can cause cell dehydration, ion accumulation, and nutritional imbalance, which can lead to cell damage. However, SACCS and FBS showed no significant difference in salinity and pH and showed similar characteristics (Fig. 1G) that facilitate the ideal culture of cells^28^. The composition and content of FBS is not completely known; it is known to be generally composed of proteins, polypeptides, hormones, metabolites, various nutrients, and inorganic substances^29^. SACCS was found to be mainly composed of proteins and minerals, which have a positive effect on cell growth^30^. Accumulation of various heavy metals such as lead, cadmium, and mercury in the marine microalgal resources has been associated with a decrease in cell viability^31,32^. However, SACCS exhibited very low levels of lead, cadmium, and mercury (Table 1). Cell number and viability were measured to analyze the effect of SACCS on the cell growth in accordance with the FBS reduction. In the F3:S7 group, there were no significant differences in cell number, cell viability, and morphological appearances of animal cells. However, in the F1:S9 group, cell growth was significantly decreased from the 9th passage onwards, and morphological changes were also detected. In the F5:S5 group, cell numbers and viability were increased significantly compared with that in the control (Fig. 3B,C). In addition, as a result of applying SACCS to HeLa cell—a cervical cancer cell, and T24 cell—a bladder cancer cell, the number of cells and cell viability were similar compared to the control group despite a replacement of up to 50–70% (Figs. S2 and S3). Therefore, the possibility that the efficacy of SACCS was applied not only to H460 but also to various cell lines was confirmed. In particular, the number of cells increased up to 20% compared to when only FBS was reduced without treatment with SACCS (Fig. S4). Therefore, SACCS exhibited a positive effect on cell proliferation. Based on these results, the cell cycle phases were analyzed in accordance with the decrease in FBS concentration. It is generally known that the cell cycle is arrested in the G_1_ phase as the FBS concentration decreases^33,34^. However, the SACCS-treated group did not show a G_1_ phase arrest and it was confirmed that the G_0_/G_1_ phase was normally operated^35–37^. When the concentration of FBS was reduced to 3%, there was no significant difference in the cell cycle. However, the G_2_/M phase increased when the concentration of FBS was reduced to 1% (Fig. 4A). Nonetheless, the inhibition of the cell cycle at G_2_/M phase is not adequate to cause cell growth arrest^35–37^. Moreover, apoptotic cells were almost non-existent, as sub-G_1_ phase was not identified in any of the experimental groups^38^. The cell cycle of F5:S5 group was similar to that of the control group, and S phase cell aggregation suggested that SACCS could increase the potential of cell proliferation^39,40^. Cell death is usually classified as necrosis and apoptosis. Recently, cell death through autophagy has also been reported^41,42^. Flow cytometry analysis of cell death in this study revealed a dramatic effect of decreasing FBS concentration. As a result of addition of SACCS instead of FBS, apoptosis was not observed in any of the experimental groups. Necrosis increased as the concentration of FBS decreased, and 47.9% of necrosis occurred at 1% FBS (Fig. 4B). However, it is known that autophagy occurs more frequently than necrosis during nutrient deficiency caused due to the lack of FBS or other nutrients^43–45^. Autophagy is similar to cell death in that it involves pyknosis and karyorrhexis, or to necrosis characterized by early disruption of the cell membrane, and is commonly known to occur before apoptosis^41,43–45^. Therefore, the cell death mechanism was identified with proteomic analysis, and it was confirmed that ubiquitin was expressed when the FBS concentration was reduced to 3% or less (Fig. 6). Ubiquitin is a regulatory protein in the ubiquitin–proteasome pathway, and it acts on cyclins, cyclin-dependent kinase inhibitors, transcription factors, cell surface receptors, antigenic peptides, and oncoproteins^46–48^. In addition, it is known to regulate important functions in damaged cells such as cell cycle, gene expression, signal transduction, cell division, cell death, immune, and inflammatory responses^46–48^. Proteins targeted by ubiquitin for degradation are first poly-ubiquitinylated, which are then recognized and degraded by the 26S proteasome complex; ubiquitin consists of a process that was free and reused, or that was cell degradation via the lysosomal/vacuolar system^46–48^. Therefore, ubiquitin accumulation is known to mediate autophagy as one of the mechanisms of cell death, and autophagy is known to be induced by FBS reduction. In the current study, we verified that cell death occurred due to autophagy because of decreased FBS. Furthermore, GO enrichment analysis confirmed that genes associated with the positive regulation of response to oxidative stress (GO: 1902884) in the biological process category was significantly higher in cells in the F1:S9 group (Fig. 5E). The effect of SACCS on the cells was induced by minimizing the FBS concentration, and it was confirmed that SACCS has a significant effect on the expression related to antioxidant function. Especially, the results of proteomic analysis confirmed that superoxide dismutase (SOD) was significantly increased with increasing concentration of SACCS, suggesting suggests that SACCS can induce cells to adapt to oxidative stress^49,50^. In addition, the F5:S5 group showed up-regulation of various genes in the biological process category, and most DEGs were involved in the retinoid metabolic process. Therefore, SACCS is thought to induce cell proliferation by the retinoid metabolic process mechanism^51,52^. In particular, it was confirmed that cell proliferation was enhanced upon treatment with 5% SACCS. The cell differentiation and antioxidant effects of these SACCS can be supported by various biological activity results using *S. maxima*. Several studies have reported that *S. maxima* affected the prevention of cardiomyoblasts in H9c2 cells, and protection of hepatic damage in a rat model^53,54^. Their effectiveness was based on strong antioxidants and various health care effects were found to be very similar to this study^55,56^. Therefore, SACCS was thought to have a synergistic effect on cell growth and proliferation, indicating new possibilities for FBS replacement. Further research is required to study the enhancement of cell growth using pretreatment methods and evaluation of the natural products included in SACCS. These are methods proposed to address challenges related to animal-derived FBS. From an economic perspective, the SACCS can be an economic alternative to other FBS alternatives because it was used without a complicated production process. In particular, it is expected to have a direct economic advantage as it reduces the use of FBS, the highest cost in cell experiments. It can also play an important role as an FBS-substitute by minimizing the environmental and social disadvantage of existing FBS extracted from animals. Therefore, it is suggested that SACCS can provide the energy source required for cell growth, proliferation, and immune activity without various growth factors provided by conventional serum-free media sources. In conclusion, our results demonstrated the possibility of using edible cyanobacterium extract as a substitute for animal serum (FBS).

## Methods

### Culture and extraction of S. maxima

Marine *Spirulina maxima* that has been originally cultivated in Korea Institute of Ocean Science & Technology (Jeju, Korea) and cultivated in vertical rounded-200 L-photobioreactor containing SOT medium with continuous aeration^19,57,58^. Cultures were grown at 25 °C under a 12:12-h light/dark photoperiod. Cells were harvested by centrifugation at 9000 rpm for 20 min (Thermo Fisher Scientific, Massachusetts, USA) and then lyophilized and stored at − 50 °C (Operon, Gimpo, South Korea)^57^.

Dried *S. maxima* powder was dissolved in distilled water (DW) at 1% (1×) and 0.5% (0.5×) (w/v), sonicated (Fig. 1A) and subjected to high temperature and pressure treatment (Fig. 1B) to disrupt the cells. The sample was centrifuged (Labogene, Daejeon, South Korea) at 9000 rpm for 20 min (Fig. 1C) and filtered through a 1-μm Whatman No. 1 filter paper (Whatman, Maidstone, England) (Fig. 1D). To separate the micro residues, the supernatant was centrifuged (Beckman Coulter, Brea, USA) at 30,000 rpm for 20 min (Fig. 1E) and filtered through a 0.2-μm filter to remove bacteria, fungi, and mycoplasma (Fig. 1F). The solutions [1% and 0.5% spirulina animal cell culture solution (SACCS)] were stored at − 20 °C until use. Under this process, the yields of SACCS extracted from *Spirulina maxima* was obtained by approximately 10%.

### Physicochemical analysis

Salinity was measured using a portable YK-31SA salt meter (Lutron, Taipei, Taiwan) and pH was measured using SevenCompact pH/Ion S220 pH meter (Mettler Toledo, Ohio, USA)^59^. We compared the pH and salinity of FBS and SACCS as well as 10% of their mixture in MEM.

### Microbial contamination assay

The prepared SACCS was tested for contamination using e-Myco polymerase chain reaction (PCR) detection kit (iNtRON Biotechnology, Seongnam, South Korea) and DiaPlexC PCR kit (AGBIO, Seoul, South Korea). PCR (Takara, Kusatsu, Japan) was performed with positive and negative controls provided in the kits, followed by electrophoresis using 1.5% agarose gel^60^.

### Component analysis

Normal components were analyzed using Korean Food Standards Codex (2015) and AOAC methods. Crude fat and protein were detected by ether extraction and Kjeldahl methods^61^, respectively. For lead and cadmium analysis, the specimen was taken out of the crucible, dried, and carbonized. After heating at 450–550 °C, the ash was wetted with water, followed by addition of 2–4 mL of hydrochloric acid and drying in an aqueous solution. Nitric acid (4%) was added to dissolve it by heating. Following filtration, the solution was adjusted to 20 mL and used as the test solution. Lead and cadmium standard solutions, test solutions, and blank were injected into ICP-OES (Varian, Palo Alto, USA) and analyzed. The standard solution was prepared by diluting a 1000 mg/L reference material with 4% nitric acid to prepare a 100 mg/L stock solution, which was then diluted with 4% nitric acid to prepare standard solutions of different concentrations. For inorganic analysis, 1 g of sample was taken in a container, carbonized, and heated at 550℃ for several hours, until white or off-white ash was obtained. The ash was sequentially decomposed using hydrochloric acid, diluted ten-fold, filtered, and quantified using an ICP analyzer (Optima 8300, Perkin Elmer, Waltham, USA)^62^.

### Cells, medium, and culture conditions

The human lung carcinoma cell line H460 was purchased from Korean cell line bank (Seoul, South Korea) and grown in RPMI 1640 medium (Gibco, Grand Island, USA) containing 10% FBS at 37 °C in 5% CO_2_. FBS100%: SACCS0% (F10:S0) solution containing only FBS was used as the control. FBS and SACCS were added into the medium at various mixing volume ratios (%, v/v): FBS50%: SACCS50% (F5:S5), FBS30%: SACCS70% (F3:S7), and FBS10%: SACCS90% (F1:S9). Each mixture was added to the cell culture medium at a 10% ratio.

### Cytotoxicity test

H460 cells (3.4 × 10^4^ cells/mL) were seeded in triplicate 96-well plates and treated with DW, FBS, 0.5×, 1×, 2×, and 3× SACCS, for 72 h. Thereafter, 10 μL WST solution (Daeillab, Seoul, South Korea) was added and cells were incubated for 3 h at 37 °C. Absorbance was recorded at 450 nm using an ELISA plate reader (Biotek, Winooski, USA).

### Cell proliferation assay

Cells were cultured in triplicate in experimental media with different F:S volume ratios, with 3 mL medium per 60-mm (ø) dish. After 72 h of growth, cells were counted and subcultured into new dishes. A total of 10 passages of such 72-h interval subculture, each in experimental media with different F:S volume ratios, were performed for the cell lines. Cell samples (20 μL) were pipetted onto a Nexcelom disposable counting chamber (Nexcelom Bioscience, LLC, USA). Total cell number was counted using the automated Cellometer Mini (Nexcelom Bioscience)^63^. Cellular morphology was analyzed using an inverted light microscope (Nikon, Tokyo, Japan).

### WST assay

For analyzing cell viability, H460 (3.4 × 10^4^ cells/mL) cells cultured in 10th generation in SACCS-containing media were seeded in 96-well plates. After 24, 48, and 72 h, absorbance was measured at 450 nm using a spectrophotometer with WST reagent^64^.

### Cell cycle analysis

Cell cycles of H460 cells cultured in SACCS and FBS were analyzed using CycleTEST PLUS DNA Reagent Kit (Becton Dickinson, CA, USA). Cells were cultured for 10 passages in a cell culture medium mixed with different F:S volume ratios (%, v/v) and seeded onto dishes at 2 × 10^5^ cells/mL and washed with PBS the following day. Cells were removed by treatment with trypsin–EDTA (Gibco) and centrifuged to remove the supernatant. Trypsin buffer was added to digest recovered cells. After 10 min, trypsin inhibitor and RNase buffer were added to degrade RNA and cells were stained with propidium iodide (PI) (selective staining of nuclear DNA) solution at 4℃ for 10 min. Cells were analyzed identically using a flow cytometer (BD biosciences, San Diego, USA)^65^.

### Apoptosis assay

H460 cells from each of the ten passages were seeded at 2 × 10^5^ cells/mL. The culture solution was removed after washing twice with PBS, and cells were centrifuged and adjusted to a density of 1 × 10^6^ cells/mL using a binding buffer. Annexin V-FITC and PI (5 µL each) were added to 100 μL of the solution. After staining at 25℃ for 15 min, 400 μL binding buffer was added and the degree of apoptosis was measured using a flow cytometer (BD biosciences)^66^.

### Transcriptomic analysis

Total RNA was extracted from cells using the RNeasy Mini kit (Qiagen, USA). Isolated RNA was stored at − 80 °C until further use. All RNA sequencing and alignment procedures were conducted by ChunLab (Seoul, South Korea). Libraries for Illumina sequencing were prepared using the TruSeq Stranded mRNA Sample Prep kit (Illumina, USA). RNA sequencing was performed on the Illumina HiSeq 2500 platform using paired-end 100-bp sequencing.

The sequence for the reference genome was retrieved from the NCBI database. Quality-filtered reads were aligned to the reference genome sequence using Bowtie2. The sequence data were normalized by Relative Log Expression method. Visualization of mapping results and analysis of differentially expressed genes (DEGs) were performed using the CLRNASeq program (ChunLab). Gene Ontology (GO) term enrichment was analyzed using GOrilla. Gorilla is publicly available as a web-based application at: http://cbl-gorilla.cs.technion.ac.il^67,68^.

### Proteomic analysis

The lysis solution constituting 7 M urea, 2 M thiourea, 4% (w/v) 3-[(3-cholamidopropy) dimethylammoniol]-1-propane-sulfonate (CHAPS), 1% (w/v) dithiothreitol, 2% (v/v) pharmalyte, and 1 mM benzamidine was added to the sample. For protein extraction, vortexing was performed for 1 h, and the supernatant after centrifugation at 25 °C and 12,000 rpm for 1 h was used for two-dimensional electrophoresis. Protein concentration was then measured^69^.

For primary isoelectric focusing (IEF), immobilized pH gradient (IPG) strips were placed in the reswelling tray with 7 M urea, 2 M thiourea, 2% CHAPS, and 1% dithiothreitol solution at room temperature for 12–16 h. IEF was performed at 20 °C using a Multipore II system (Amersham Biosciences, Little Chalfont, England). The conditions were set such that 3 h was required to reach 3500 V from 150 V, followed by 26 h at 3500 V, and finally to 96 kVh. IPG strips were incubated with equilibration buffer (50 mM Tris–Cl, pH 6.8, 6 M urea, 2% SDS, 30% glycerol) containing 1% DTT for 10 min before secondary SDS-PAGE, and then incubated for 10 min with equilibration buffer containing iodoacetamide. Equilibration-completed strips were arrayed on SDS-PAGE gels (20 × 24 cm, 10–16%) and developed to a final 1.7 kVh at 20 °C using a Hoefer DALT 2D system (Amersham Biosciences). The gel was visualized after 2D electrophoresis using colloidal Coomassie brilliant blue (CBB) staining according to a previous method^70^; glutaraldehyde treatment was omitted for protein identification by mass spectrometry. Colloidal CBB-stained 2D gels were scanned using a DuoScan T1200 scanner (AGFA, Mortsel, Belgium).

Quantitative analysis of protein spots from scanned images was performed using PDQuest software (version 7.0, Bio-Rad, Hercules, USA). Quantity of each spot was normalized to the intensity of the total valid spots and protein spots showing more than two-fold significant changes in expression compared to the control were selected.

### Statistical analysis

All results are represented as the mean ± standard deviation. Differences between groups were assessed by two-way ANOVA with Dunnett’s post-test using GraphPad Prism (ver. 8.1.1, San Diego, USA). A *p* value < 0.05 was considered significant.

## Supplementary Information

Supplementary information.

## References

[1] Maurer, H. R. Towards chemically-defined, serum-free media for mammalian cell culture. In: *Animal Cell Culture a practical approach*. (ed Freshney, R. I.) 13–31 (IRL Press, Oxford, 1986).

[2] Klein R, Dumble L (1993). Transmission of Creutzfeldt-Jakob disease by blood transfusion. The Lancet.

[3] Jochems CE, Van Der Valk JB, Stafleu FR, Baumans V (2002). The use of fetal bovine serum: ethical or scientific problem?. ATLA-NOTTINGHAM.

[4] Eloit M (1999). Risks of virus transmission associated with animal sera or substitutes and methods of control. Dev. Biol. Stand..

[5] Shah G (1999). Why do we still use serum in the production of biopharmaceuticals?. Dev. Biol. Stand..

[6] Wessman S, Levings R (1999). Benefits and risks due to animal serum used in cell culture production. Dev. Biol. Stand..

[7] Gstraunthaler G (2003). Alternatives to the use of fetal bovine serum: Serum-free cell culture. ALTEX-Altern. Anim. Exp..

[8] Fang C-Y, Wu C-C, Fang C-L, Chen W-Y, Chen C-L (2017). Long-term growth comparison studies of FBS and FBS alternatives in six head and neck cell lines. PLoS ONE.

[9] Zamansky GB, Arundel C, Nagasawa H, Little JB (1983). Adaptation of human diploid fibroblasts in vitro to serum from different sources. J. Cell Sci..

[10] Paranjape, S. Goat serum: an alternative to fetal bovine serum in biomedical research (2004). 15274477

[11] Franke J, Abs V, Zizzadoro C, Abraham G (2014). Comparative study of the effects of fetal bovine serum versus horse serum on growth and differentiation of primary equine bronchial fibroblasts. BMC Vet. Res..

[12] Ziegler A (2016). Equine dendritic cells generated with horse serum have enhanced functionality in comparison to dendritic cells generated with fetal bovine serum. BMC Vet. Res..

[13] Ciferri O (1983). Spirulina, the edible microorganism. Microbiol. Rev..

[14] Gershwin ME, Belay A (2007). Spirulina in Human Nutrition and Health.

[15] Venkataraman LV (1997). Spirulina platensis (Arthrospira): physiology, cell biology and biotechnologym, edited by Avigad Vonshak. J. Appl. Phycol..

[16] Belay A, Ota Y, Miyakawa K, Shimamatsu H (1993). Current knowledge on potential health benefits of Spirulina. J. Appl. Phycol..

[17] Blinkova, L., Gorobets, O. & Baturo, A. Biological activity of Spirulina Zh Mikrobiol Epidemiol Immunobiol. 2001 Mar-Apr;(2): 114–8. Review. In this review information of Spirulina platensis (SP), a blue-green alga (photosynthesizing cyanobacterium) having diverse biological activity is presented. Due to high content of highly. *Zh Mikrobiol. Epidemiol. Immunobiol.* **2**, 114–118 (2001). 11548244

[18] Khan Z, Bhadouria P, Bisen P (2005). Nutritional and therapeutic potential of Spirulina. Curr. Pharm. Biotechnol..

[19] Oh S-H, Ahn J, Kang D-H, Lee H-Y (2011). The effect of ultrasonificated extracts of Spirulina maxima on the anticancer activity. Mar. Biotechnol..

[20] Gutiérrez-Rebolledo GA (2015). Antioxidant effect of Spirulina (Arthrospira) maxima on chronic inflammation induced by Freund's complete adjuvant in rats. J. Med. Food.

[21] Ponce-Canchihuamán JC, Pérez-Méndez O, Hernández-Muñoz R, Torres-Durán PV, Juárez-Oropeza MA (2010). Protective effects of Spirulina maxima on hyperlipidemia and oxidative-stress induced by lead acetate in the liver and kidney. Lipids Health Dis..

[22] Lee J (2017). Spirulina extract enhanced a protective effect in type 1 diabetes by anti-apoptosis and anti-ROS production. Nutrients.

[23] Koh E-J (2017). Spirulina maxima extract prevents neurotoxicity via promoting activation of BDNF/CREB signaling pathways in neuronal cells and mice. Molecules.

[24] De Oliveira M, Monteiro M, Robbs P, Leite S (1999). Growth and chemical composition of Spirulina maxima and Spirulina platensis biomass at different temperatures. Aquacult. Int..

[25] Schafer K (1998). The cell cycle: A review. Vet. Pathol..

[26] Drexler HG, Uphoff CC (2002). Mycoplasma contamination of cell cultures: Incidence, sources, effects, detection, elimination, prevention. Cytotechnology.

[27] Folmsbee M, Howard G, McAlister M (2010). Nutritional effects of culture media on mycoplasma cell size and removal by filtration. Biologicals.

[28] Yi X, Sun X, Zhang Y (2004). Effects of osmotic pressure on recombinant BHK cell growth and von willebrand factor (vWF) expression. Process Biochem..

[29] Van der Valk J (2010). Optimization of chemically defined cell culture media–replacing fetal bovine serum in mammalian in vitro methods. Toxicol. In Vitro.

[30] Rao M (1994). Effects of vitamin/mineral supplementation on the proliferation of esophageal squamous epithelium in Linxian China. Cancer Epidemiolo. Prevent. Biomark..

[31] Fischer AB, Škreb Y (2001). In vitro toxicology of heavy metals using mammalian cells: an overview of collaborative research data. Arhiv za Higijenu Rada i Toksikologiju.

[32] Rosko, J. J. & Rachlin, J. W. The effect of cadium, copper, mercury, zinc and lead on cell division, growth, and chlorophyll a content of the chlorophyte Chlorella vulgaris. *Bull. Torrey Bot. Club* **104**, 226–233 (1977).

[33] Khammanit R, Chantakru S, Kitiyanant Y, Saikhun J (2008). Effect of serum starvation and chemical inhibitors on cell cycle synchronization of canine dermal fibroblasts. Theriogenology.

[34] Rashid MU, Coombs KM (2019). Serum-reduced media impacts on cell viability and protein expression in human lung epithelial cells. J. Cell. Physiol..

[35] Wang Z (2008). Methylseleninic acid inhibits microvascular endothelial G1 cell cycle progression and decreases tumor microvessel density. Int. J. Cancer.

[36] Kaeck M (1997). Differential induction of growth arrest inducible genes by selenium compounds. Biochem. Pharmacol..

[37] Sinha R, Said T, Medina D (1996). Organic and inorganic selenium compounds inhibit mouse mammary cell growth in vitro by different cellular pathways. Cancer Lett..

[38] Shu C-H, Yang W, Shih Y-L, Kuo M-L, Huang T-S (1997). Cell cycle G2/M arrest and activation of cyclin-dependent kinases associated with low-dose paclitaxel-induced sub-G1 apoptosis. Apoptosis.

[39] Wang Y (2008). Embryonic stem cell–specific microRNAs regulate the G1-S transition and promote rapid proliferation. Nat. Genet..

[40] Krueger SA, Wilson GD (2011). Cancer Cell Culture.

[41] Marino G, Niso-Santano M, Baehrecke EH, Kroemer G (2014). Self-consumption: the interplay of autophagy and apoptosis. Nat. Rev. Mol. Cell Biol..

[42] He C, Klionsky DJ (2009). Regulation mechanisms and signaling pathways of autophagy. Ann. Rev. Genet..

[43] Huang Y (2018). Serum starvation-induces down-regulation of Bcl-2/Bax confers apoptosis in tongue coating-related cells in vitro. Mol. Med. Rep..

[44] Kristensen AR (2008). Ordered organelle degradation during starvation-induced autophagy. Mol. Cell. Proteom..

[45] Sandag Z (2020). Inhibitory role of TRIP-Br 1/XIAP in necroptosis under nutrient/serum starvation. Mol. Cells.

[46] Grumati P, Dikic I (2018). Ubiquitin signaling and autophagy. J. Biol. Chem..

[47] Ciechanover A (1998). The ubiquitin–proteasome pathway: On protein death and cell life. EMBO J..

[48] Kornitzer D, Ciechanover A (2000). Modes of regulation of ubiquitin-mediated protein degradation. J. Cell. Physiol..

[49] Chu W-L, Lim Y-W, Radhakrishnan AK, Lim P-E (2010). Protective effect of aqueous extract from Spirulina platensis against cell death induced by free radicals. BMC Complem. Altern. Med..

[50] Alevriadou BR, Shanmughapriya S, Patel A, Stathopulos PB, Madesh M (2017). Mitochondrial Ca2+ transport in the endothelium: Regulation by ions, redox signalling and mechanical forces. J. R. Soc. Interface.

[51] Blomhoff R, Blomhoff HK (2006). Overview of retinoid metabolism and function. J. Neurobiol..

[52] Gudas LJ (1992). Retinoids, retinoid-responsive genes, cell differentiation, and cancer. Cell Growth Differ..

[53] Jadaun P, Yadav D, Bisen PS (2018). Spirulina platensis prevents high glucose-induced oxidative stress mitochondrial damage mediated apoptosis in cardiomyoblasts. Cytotechnology.

[54] Jatav SK (2014). Spirulina maxima protects liver from Isoniazid and Rifampicin drug toxicity. J. Evid. Based Complem. Altern. Med..

[55] Jarouliya U, Anish ZJ, Kumar P, Bisen P, Prasad G (2012). Alleviation of metabolic abnormalities induced by excessive fructose administration in Wistar rats by Spirulina maxima. Indian J Med. Res..

[56] Kulshreshtha A, Jarouliya U, Bhadauriya P, Prasad G, Bisen P (2008). Spirulina in health care management. Curr. Pharm. Biotechnol..

[57] Choi WY, Kang DH, Lee HY (2013). Enhancement of immune activation activities of Spirulina maxima grown in deep-sea water. Int. J. Mol. Sci..

[58] Kim, T. *et al.* Cultivating spirulina maxima: Innovative approaches. *Cyanobacteria*, 61 (2018).

[59] Boström M, Craig VS, Albion R, Williams DR, Ninham BW (2003). Hofmeister effects in pH measurements: role of added salt and co-ions. J. Phys. Chem. B.

[60] Ashbolt NJ (2004). Microbial contamination of drinking water and disease outcomes in developing regions. Toxicology.

[61] Bradstreet RB (1954). Kjeldahl method for organic nitrogen. Anal. Chem..

[62] Al-Dhabi NA (2013). Heavy metal analysis in commercial Spirulina products for human consumption. Saudi J. Biol. Sci..

[63] Gordobil O (2018). Potential use of kraft and organosolv lignins as a natural additive for healthcare products. RSC Adv..

[64] Peskin AV, Winterbourn CC (2000). A microtiter plate assay for superoxide dismutase using a water-soluble tetrazolium salt (WST-1). Clin. Chim. Acta.

[65] Hartwell LH, Kastan MB (1994). Cell cycle control and cancer. Science.

[66] Lowe SW, Lin AW (2000). Apoptosis in cancer. Carcinogenesis.

[67] Eden E, Navon R, Steinfeld I, Lipson D, Yakhini Z (2009). GOrilla: a tool for discovery and visualization of enriched GO terms in ranked gene lists. BMC Bioinform..

[68] Eden E, Lipson D, Yogev S, Yakhini Z (2007). Discovering motifs in ranked lists of DNA sequences. PLoS Comput. Biol..

[69] Bradford MM (1976). A rapid and sensitive method for the quantitation of microgram quantities of protein utilizing the principle of protein-dye binding. Anal. Biochem..

[70] Oakley BR, Kirsch DR, Morris NR (1980). A simplified ultrasensitive silver stain for detecting proteins in polyacrylamide gels. Anal. Biochem..

