# Supplementary material for: Marine cyanobacterium Spirulina maxima as an alternate to the animal cell culture medium supplement
Source: Sci Rep. 2021 Mar 1;11:4906. doi: 10.1038/s41598-021-84558-2 (PMC7921123; doi:10.1038/s41598-021-84558-2)

Marine cyanobacterium *Spirulina maxima* as an alternate to the animal cell culture medium supplement

Younsik Jeong[1], Woon-Yong Choi[1], Areumi Park[1], Yeon-Ji Lee[1], Youngdeuk Lee[1], Gun-Hoo Park[1,2], Su-Jin Lee[1], Won-Kyu Lee[1,3], Yong-Kyun Ryu[1,3] & Do-Hyung Kang[1,3*]

[1]Jeju Marine Research Center, Korea Institute of Ocean Science and Technology (KIOST), Jeju, Republic of Korea. [2]School of Pharmacy, Sungkyunkwan University, Seoul, Republic of Korea. [3]Department of Ocean Science, University of Science and Technology (UST), Jeju, Republic of Korea.

Supplementary Figure S1. Native gel of contamination test on SACCS. SACCS was tested for mycoplasma contamination using e-Myco™ PCR detection kit and fungal and bacterial contamination using DiaPlexC™ PCR kit.

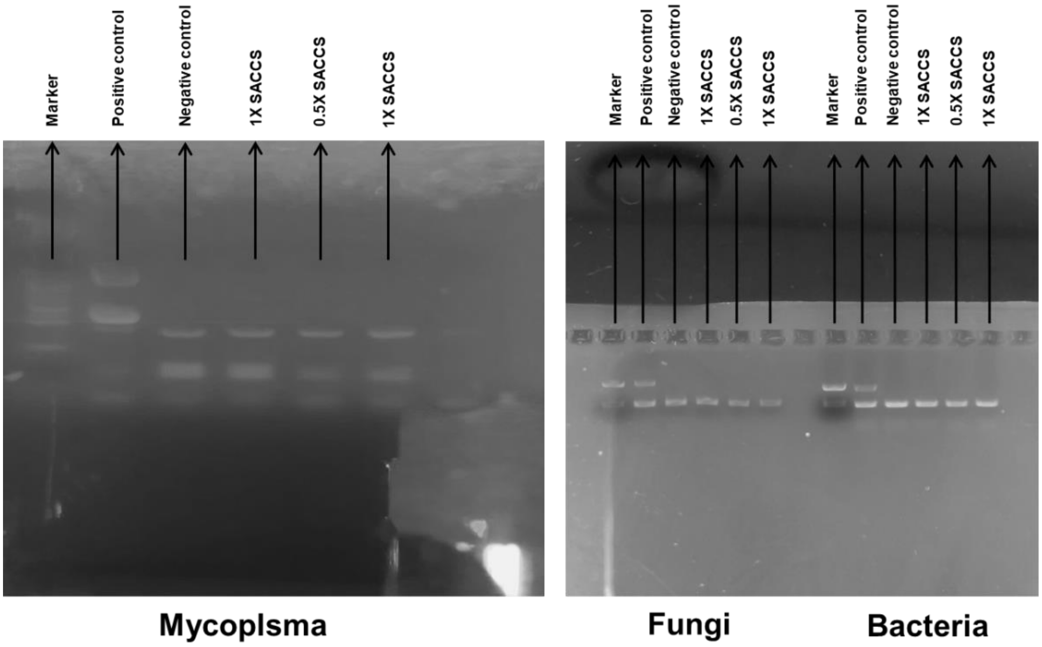

Supplementary Figure S2. Evaluation of FBS substitution efficacy of SACCS in HeLa and T24 cells. Comparison of morphology and metabolic proliferation activities of the two cells (HeLa (A and C) and T24 (B and D) cells) in control medium (FBS) and SACCS-substituted medium under different ratios between 1× SACCS (A and B) and half diluted 0.5× SACCS (C and D). Scale bar: 100 μm. Data are expressed as mean values ± SD of triplicate independent experiments.

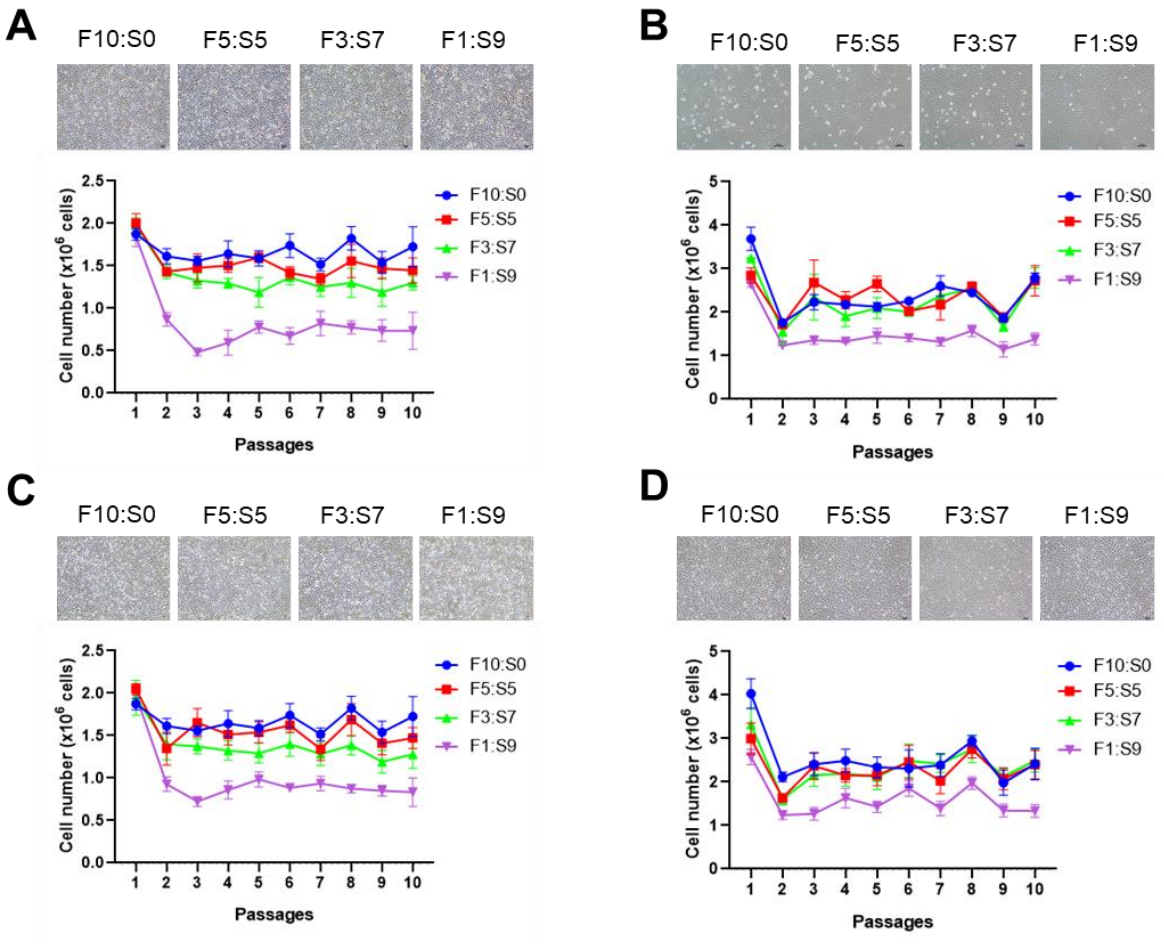

Supplementary Figure S3. Variation in cell viability with FBS substitution of SACCS in HeLa and T24 cells. Viability of HeLa (A and C) and T24 (B and D) cells cultured for 10 passages in control medium (FBS) and SACCS-substituted medium under different ratios between 1× SACCS (A and B) and half diluted 0.5× SACCS (C and D). Data are expressed as mean values ± SD of triplicate independent experiments. * $P < 0.05$; ** $P < 0.01$; ***$P < 0.001$ compared with cells cultured in FBS.

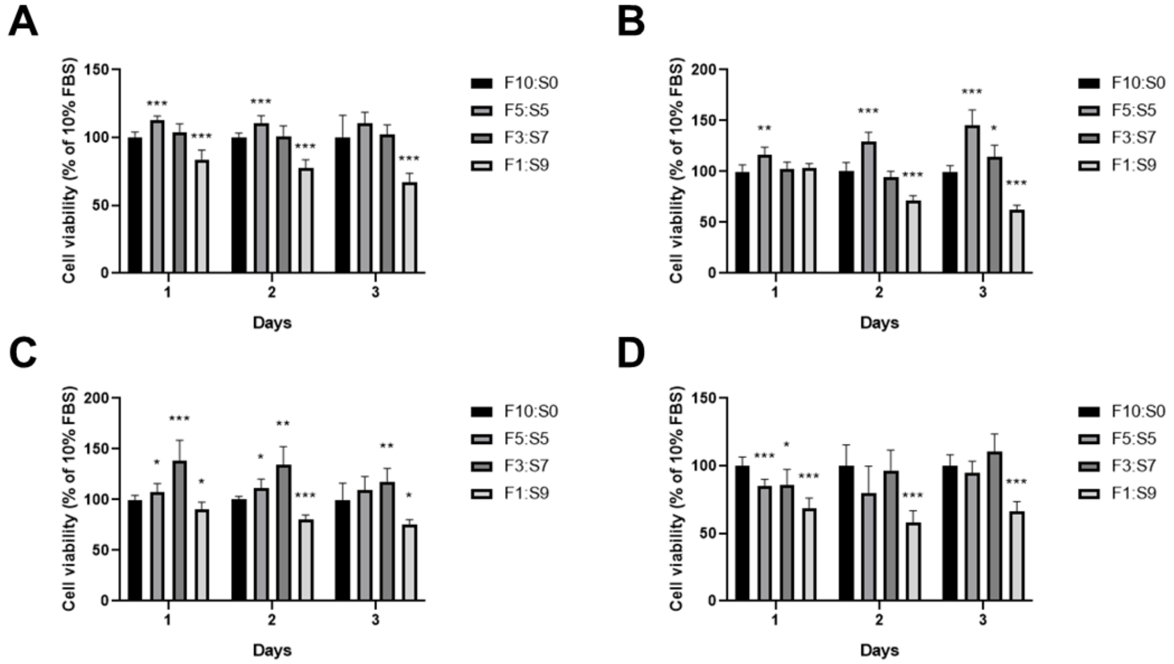

Supplementary Figure S4. Measurement of cell number with changes in FBS ratio. Comparison of cell proliferation activities of the H460, HeLa and T24 cells in F5:S0, F3:S0, and F1:S0 medium. Data are expressed as mean values ± SD of triplicate independent experiments.

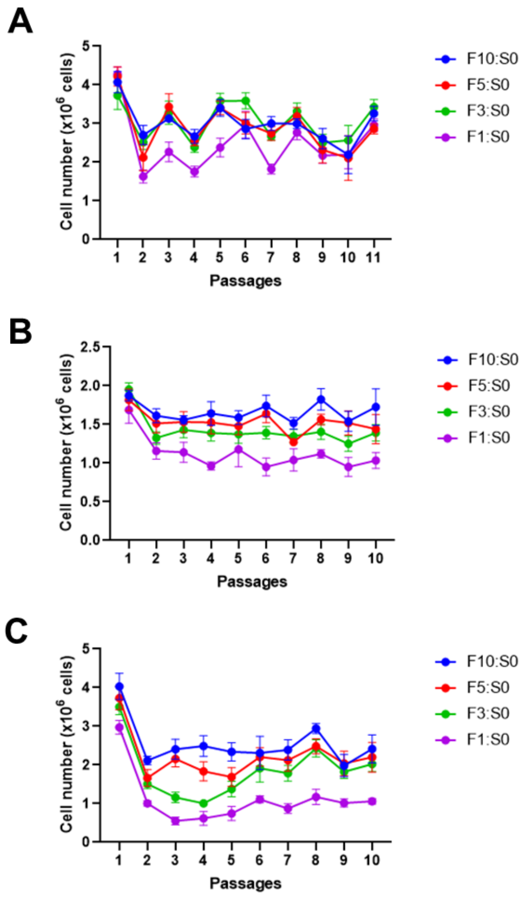

Supplement: Supplementary file 1 — Supplementary information. [file 41598_2021_84558_MOESM1_ESM.pdf]
